# Autogenous osteochondral graft transplantation for steroid-induced osteonecrosis of the femoral condyle: A report of three young patients

**DOI:** 10.1186/1758-2555-4-13

**Published:** 2012-04-26

**Authors:** Norifumi Fujita, Tomoyuki Matsumoto, Seiji Kubo, Takehiko Matsushita, Kazunari Ishida, Yuichi Hoshino, Koji Nishimoto, Masahiro Kurosaka, Ryosuke Kuroda

**Affiliations:** 1 Department of Orthopaedic Surgery, Kobe University Graduate School of Medicine, 7-5-2 Kusunoki-cho, Chuo-ku, Kobe, 650-0017, Japan

**Keywords:** Steroid-induced osteonecrosis, Osteochodral autograft transplantation, Mosaicplasty, Femoral condyle

## Abstract

Steroid-induced osteonecrosis of the femoral condyle is a relatively uncommon condition and is often difficult to select appropriate treatment especially in young patients. Three young men (aged 25, 18, and 24) presented with severe pain and dysfunction of the knee diagnosed as steroid-induced osteonecrosis of the femoral condyle by magnetic resonance imaging (MRIs). Full-thickness cartilage defects sized 20 × 10, 15 × 10, and 30 × 20 mm respectively were classified as International Cartilage Repair Society Grade IV lesions and treated with osteochondral autograft transplantation. They were treated successfully with osteochondral autograft transplantation certificated by post-operative MRI and second look arthroscopy.

## Background

Steroid-induced osteonecrosis of the femoral condyle is a relatively uncommon condition and its clinical course and established treatment remain controversial, mainly because of the limited number of cases [1,2]. Steroid-induced osteonecrosis is a debilitating clinical problem that frequently occurs in younger patients and is associated with a variety of disease states. Progression of necrosis may lead to subchondral bone collapse, joint incongruity, subsequent joint destruction, and the need for surgical treatment. A variety of surgical techniques have been performed such as high tibial osteotomy, unilateral knee arthroplasty, and total knee arthroplasty [3]. However, these treatment options are usually considered over indications and not the ideal choices for younger patients from the viewpoint of articular cartilage regeneration and restoration. Recently, autogenous osteochondral graft has gained clinical popularity as a treatment for spontaneous osteonecrosis and cartilage defect [4-6]. Osteochondral autograft transplantation enables the restoration of articular cartilage and cartilage regeneration is expected.

We encountered three young patients with steroid-induced osteonecrosis of the femoral condyle and performed osteochondral autograft transplantations. In this report, we outline the cases and give an overview of their treatment.

## Case presentation

### Case 1

**Figure 1:**
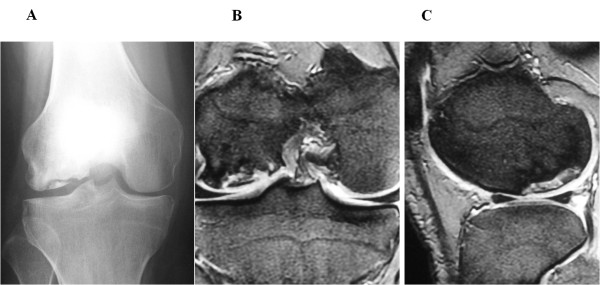
**Preoperative findings in case 1. (A)** Anteroposterior radiograph reveals an osteonecrotic lesion in the right lateral femoral condyle. **(B,C)** Coronal and saggital MRI shows an osteonecrotic lesion with high signal change on the T2-weighted image in the right lateral femoral condyle.

**Figure 2:**
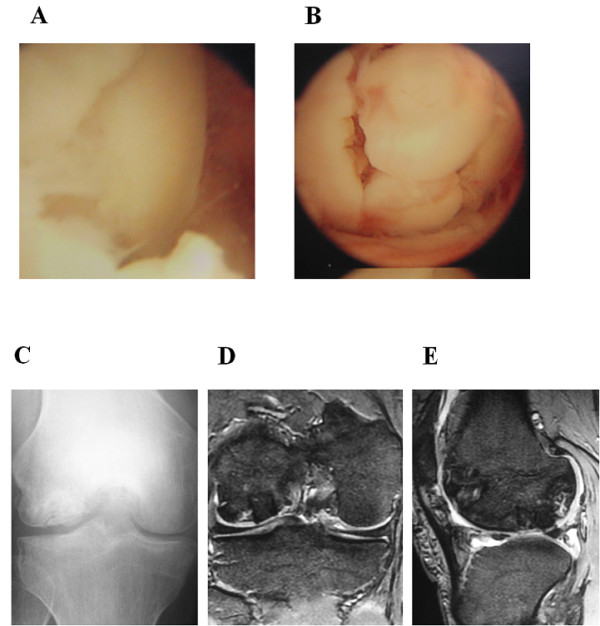
**Operative and postoperative findings in case 1. (A)** Arthroscopy showing the lesion of the osteonecrosis at the medial condyle of the femur demonstrating the anterior margin of the femoral condylar defect. **(B)** Osteochondral plugs are grafted into the recipient site. **(C)** Anteroposterior radiograph taken one year after surgery shows less irregularity of the subchondral bone of the lateral femoral condyle and a larger sclerotic area. **(D, E)** Coronal and saggital MRIs taken one year after surgery also show less irregularity of the subchondral bone and the reduction of the osteonecrotic area as compared with the preoperative MR images (Figure 1B,C), although a high signal area remains slightly in the lateral and posterior aspect of the lesion.

A 25-year-old man suffering from severe right knee pain presented at our institution. He had received steroid-pulse therapy several times for nephrotic syndrome and was subsequently diagnosed with steroid-induced osteonecrosis of the lateral femoral condyle. The preoperative X-ray and MRI showed an osteochondral defect of the lateral femoral condyle (Figure 1A-C). Arthroscopic findings showed his cartilage defect was classified as International Cartilage Repair Society Grade IV lesion. Osteochondral autograft transplantation was performed using the Arthrex osteochondral autograft transfer system (Arthrex, Naples, Fla.). Bone plugs 9 mm in diameter and 15 mm in depth were removed from the lesion (recipient site). Then two osteochondral plugs 1.0 mm oversized in diameter and of the same length were harvested from the lateral patello-femoral joint surface of the ipsilateral knee and transplanted into the recipient site measuring 20 × 10 mm using the press-fit technique (Figure 2A-B). Postoperatively, the patient received continuous passive motion (CPM), but remained non-weight bearing for 4 weeks. Quadriceps and hamstring strengthening exercises were encouraged. The patient had to keep taking steroid after the operation in order to control primary disease. Postoperatively, no further clinical symptoms occurred after surgery and his Lysholm score had improved from 67 to 100 four year after surgery. X-rays and MRIs one year after surgery showed the restoration of the articular cartilage surface and good engraftment of the graft (Figure 2C-E). We did not find any abnormality in the donor site condition.

### Case 2

**Figure 3:**
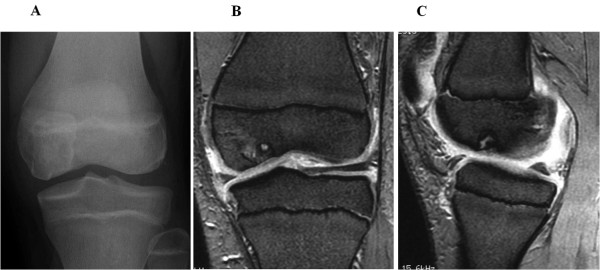
**Preoperative findings in case 2. (A)** Anteroposterior radiograph reveals an osteonecrotic lesion in the left medial femoral condyle. **(B,C)** Coronal and saggital MRI shows an osteonecrotic lesion with high signal change on the T2-weighted image in the left medial femoral condyle.

**Figure 4:**
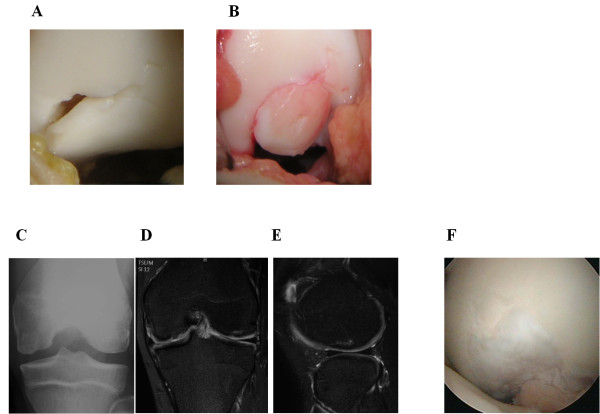
**Operative and postoperative findings in case 2. (A)** Arthroscopy shows that the free margin of a chondral fragment still partially attached to the femoral condyle posteriorly. The anterior margin of the femoral condylar defect is seen. **(B)** An osteochondral plug is grafted into the recipient site. **(C)** Anteroposterior radiograph taken two year after surgery shows less irregularity of the subchondral bone of the medial femoral condyle. **(D, E)** MRI taken two years after surgery shows the restoration of the articular cartilage surface and good engraftment of the graft. **(F)** Second look arthroscopic finding of the transplanted site demonstrates that the lesion is covered with cartilageous tissue.

An 18-year-old man suffering from severe left knee pain had received a renal transplant because of focal segmental glomerulosclerosis and was subsequently diagnosed with steroid-induced osteonecrosis of the medial femoral condyle. The preoperative X-ray and MRI showed an osteochondral defect of the medial femoral condyle (Figure 3A-C). Preoperatively, the cartilage defect was predicted to be classified as an International Cartilage Repair Society Grade IV lesion and arthroscopic evaluation demonstrated the same grade lesion of the femoral medial condyle (Figure 4A). Osteochondral graft transplantation was performed utilizing the Arthrex osteochondral autograft transfer system (Arthrex, Naples, Fla.). A bone plug 9 mm in diameter and 13 mm in depth was removed from the lesion (recipient site). Then an osteochondral plug 1.0 mm oversized in diameter and of the same length was harvested from the lateral patello-femoral joint surface of the ipsilateral knee and transplanted into the recipient site measuring 10 × 15 mm using the press-fit technique. However, the extended lesion partially to the non-weight bearing deep area from the transplanted site made it difficult to perform grafting even during deep knee flexion. Thus we only stimulated the bone marrow by using the microfracture technique at the lesion (Figure 4B). Postoperative managements were performed in the same manner as in Case 1. The patient had to keep taking steroid after the operation in order to control primary disease. X-rays and MRIs taken two years after surgery showed the restoration of the articular cartilage surface and good engraftment of the graft (Figure 4C-E). A second-look arthroscopy performed two years after surgery showed that the lesion was covered with cartilageous tissue even though a part of the non-grafting site at the posterior aspect of the defect exhibited only fibrous tissue coverage (Figure 4F). The surface of the articular cartilage was smooth both at the recipient and donor sites. We did not find any abnormality in the donor site condition. The patient had no pain and no restriction in daily activities and the Lysholm score had improved from 68 to 100 five years after surgery.

### Case 3

**Figure 5:**
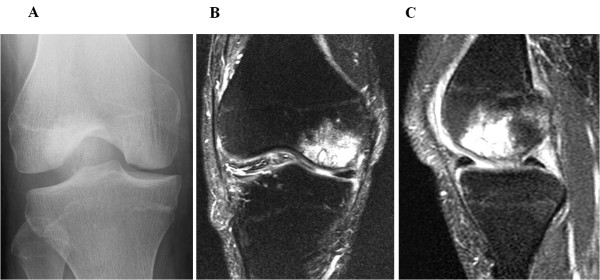
**Preoperative findings in case 3. (A)** Anteroposterior radiograph reveals an osteonecrotic lesion in the right medial femoral condyle. **(B,C)** Coronal and saggital MRI shows an osteonecrotic lesion with high signal change on the T2-weighted fat suppression image in the right medial femoral condyle.

**Figure 6:**
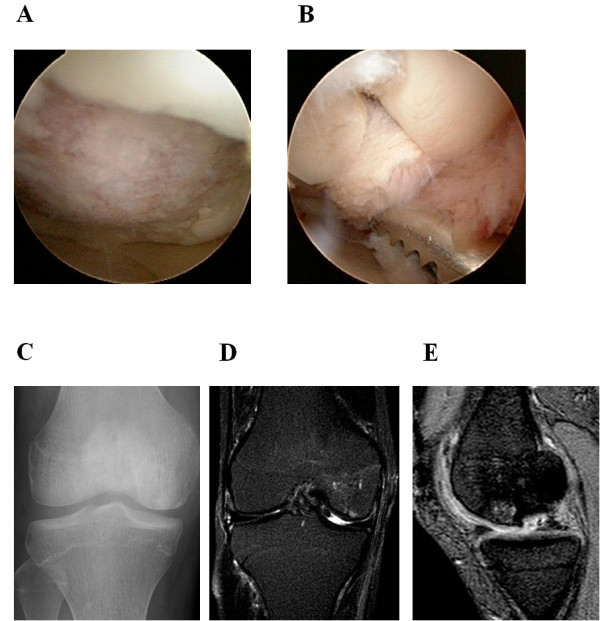
**Operative and postoperative findings in case 3. (A)** Arthroscopy showing the lesion of the osteonecrosis at the medial condyle of the femur demonstrating the anterior wide margin of the femoral condylar defect. **(B)** Osteochondral plugs are grafted into the recipient site. **(C)** Anteroposterior radiograph taken six months after surgery shows less irregularity of the subchondral bone of the medial femoral condyle. **(D, E)** MRI taken six months after surgery shows the restoration of the articular cartilage surface and good engraftment of the graft.

A 24-year-old man suffering from severe right knee pain had received a renal transplant because of focal segmental glomerulosclerosis and was subsequently diagnosed with steroid-induced osteonecrosis of the medial femoral condyle. The preoperative X-ray and MRI showed an osteochondral defect of the medial femoral condyle (Figure 5A-C). Following the arthroscopic confirmation of International Cartilage Repair Society Grade IV lesion, osteochondral graft transplantation was performed utilizing the Arthrex osteochondral autograft transfer system (Arthrex, Naples, Fla.). Bone plugs 9 mm in diameter and 13 mm in depth were removed from the lesion (recipient site). Then osteochondral plugs 1.0 mm oversized in diameter and of the same length were harvested from the lateral patello-femoral joint surface of the ipsilateral knee and transplanted into the recipient site measuring 30 × 20 mm using the press-fit technique. However, similar to Case 2, the lesion in the deep posterior site partially extended to the non-weight-bearing area from the transplanted site made it difficult to perform fully coverage with grafting. Thus we only performed bone marrow stimulation using the microfracture technique (Figure 6A-B). Postoperative management was performed in the same way as Case 1 and 2. The patient had to keep taking steroid after the operation in order to control primary disease. X-rays and MRIs six months after surgery showed the restoration of the articular cartilage surface and the good engraftment of the graft (Figure 6C-E). We did not find any abnormality in the donor site condition. The patient had no pain and no restriction in daily activities and the Lysholm score had improved from 64 to 100 six months after surgery.

## Discussion

As steroid-induced osteonecrosis of the femoral condyle is a relatively rare disease, literature regarding its treatment and histology is sparse [1,2], and there are no prospective randomized trials comparing treatment options. Thus, especially in young patients, surgeons often have difficulties in selecting an appropriate treatment. Several reports have described surgical procedures for the treatment of steroid-induced osteonecrosis of the femoral condyle [7-10]. However, there are no clear indications established for these surgical methods or for conservative treatment. As for the primary spontaneous osteonecrosis of the knee (SONK), P.J. Yates et al. reported that middle aged patients presenting with primary SONK not visible on plain radiographs, can expect a relatively rapid and complete recovery with a simple non-operative treatment [11]. Lotke et al. described that conservative treatment will do well if the size of the lesion is small (less than 45% of the condylar width, or less than 3.5 square centimeters), however, thereafter degenerative changes will develop in almost all patients[12]. However there are few literatures described of clinical course and prognosis of steroid-induced osteonecrosis of the femoral condyle. Prosthetic replacement remains the most predictable modality for treating the advanced disease, however as compared to osteoarthritis, the complication rate may be higher and the ultimate success rate slightly lower [3,13]. In addition, for young patients such as our patients, this option may be considered an over indication and is not the ideal from the view point of articular cartilage regeneration and restoration. There are other choices of treatment in clinical use such as debridement [14], abrasion chondroplasty[15], subchondral drilling [16], and microfracture [17]. These methods are based on the perforation of the underlying subchondral bone and enable the migration of pluripotent mesenchymal stem cells from the bone marrow into the defect zone. Wiedel et al. reported his experience with arthroscopic evaluation and treatment of ten knees with steroid-induced osteonecrosis of the knee [9]. He suggested that arthroscopic debridement provides reasonable symptomatic relief, allowing the patients to return to activities of daily living. However these methods have led to the formation of fibrocartilagious scar tissue with structural and biomechanical properties that are inferior to those of hyaline cartilage [16,18,19]. To this end, osteochondral allo/autografting has recently received much attention as an alternative approach for repairing joint surfaces. Osteochondral allografting is one of the available techniques for transplantation of osteochondral bone, however it has the potential risks of disease transmission or immune graft rejection [20-23]. Therefore, in three cases presented here, we indicated the osteochondral autografting for the young patients with steroid-induced osteonecrosis of the femoral condyle who were resistant to conservative treatments including restricted weight-bearing with crutches, nonsteroidal anti-inflammatory drugs, and intra-articular injection of hyaluronic acid.

Recently, use of an autogenous osteochondral graft has gained in clinical popularity because of its technical feasibility and cost effectiveness. Animal and clinical studies have shown that osteochondral plugs maintain hyaline cartilage coverage over the subchondral bone [24]. However, there are few studies regarding autogenous osteochondral graft transplantation for steroid-induced osteonecrosis of the femoral condyle. Here we reported three cases of steroid-induced osteonecrosis of the femoral condyle treated successfully with autogenous osteochondral graft transplantation. Nakagawa et al. also previously reported a case of steroid-induced osteonecrosis of the femoral condyle measuring 10 cm^2^ treated by osteochondral graft transplantation with a satisfactory result [4]. In contrast, Ching-Jen Wang et al. reported a poor result in one patient with a 6 cm^2^ defect undergoing osteochondral autograft for steroid-induced osteonecrosis of the femoral condyle [6]. In our cases, the average defect size was 2.83 cm^2^ (range 1.5 to 5.0 cm^2)^ and all patients received satisfactory results. One of the factors for the success of autogenous osteochondral mosaicplasty is the size of osteochondral articular cartilage defect. Hangody et al. reported that the defect size between 1 and 4 cm^2^ is the promising factor for the success of the procedure. Lane et al. suggested that because of the difficulty of matching the topography of recipient and donor joint surfaces, the amount of tissue that can be successfully transferred in most surgeons’ hands is limited to less than 2 cm^2^. Since autogenous osteochondral graft transplantation is a surgical procedure with free bone graft transplantation, the engraftment of the transplanted graft is an important issue, however no detailed analysis of this issue has been reported in cases of surgically treated steroid-induced osteonecrosis of the femoral condyle. Our study has shown that the transplanted grafts remained viable up to two years after surgeries based on MRIs examination and arthroscopic evaluation. However, the lesion in case 2 was partially associated with fibrous tissue formation according to a second-look arthroscopic evaluation despite a good clinical outcome. In this patient we were unable to completely perform osteochondral autograft transplantation and only performed microfracture at the deep posterior part of the lesion because of the location of the lesion. In case 3, similarly, the posterior aspect of the lesion was difficult to reach for grafting even with the knee fully flexed. A previous study suggested that osteochondral graft stability plays an important role in preserving the histologic properties of the cartilage [25]. As a result, in our cases, appropriate press-fit techniques to the peripheral lesion may have led to the reconstruction of smooth articular cartilage despite fibrous tissue coverage at the non-grafting site. However, our cases need to be followed for a longer period since our follow-up period is not long enough for the evaluation of the graft integrity, especially under steroid induced pathology.

## Conclusions

Osteochondral autograft transplantation for focal full thickness articular cartilage defects induced by steroids achieved excellent clinical results in three knees. Even though the lesion of osteonecrosis extended to the non-weight-bearing deep posterior site where it was difficult to perform the perpendicular graft transplantation and we only did the bone marrow stimulation with microfracture technique, the lesion including the non-weight-bearing area was successfully healed with smooth articular surface and fibrous tissue, leading to excellent clinical outcomes in the short-term follow up. We believe the osteochondral graft has the potential to prevent or delay the development of degenerative changes of the knee and is a good treatment method for focal steroid-induced osteonecrosis of the femoral condyle.

## Consent

Informed consent was obtained from the patient for publication of this case report and any accompanying image.

## Competing interests

None of the authors received financial support for this study.

## Authors’ contribution

YH, KN, MK and RK carried out the surgical treatment and NF, TM, SK, TM, KI and RK discussed the results and commented on the manuscript. All authors have read and proved the final manuscript.
